# Fluopicolide-Induced Oxidative Stress and DNA Damage in the Earthworm *Eisenia foetida*

**DOI:** 10.3390/toxics11100808

**Published:** 2023-09-25

**Authors:** Shengfang Wen, Youwei Wang, Xueting Wang, Chang Liu, Yannan Xue, Chao Liu, Jinhua Wang, Xiaoming Xia

**Affiliations:** 1 College of Plant Protection, Shandong Agricultural University, Tai’an 271018, China; wsf0887@163.com (S.W.); wywei2019@163.com (Y.W.); wxt111007@163.com (X.W.); liuchang971001@163.com (C.L.); xyn81926@163.com (Y.X.); liuchao19950519@163.com (C.L.); 2 College of Resources and Environment, Shandong Agricultural University, Tai’an 271018, China; wjh@sdau.edu.cn

**Keywords:** fluopicolide, Eisenia foetida, oxidative stress, DNA damage

## Abstract

Fluopicolide is a new benzamide fungicide with a unique mechanism of action and is toxic to some non-target organisms. However, there is a lack of research on the chronic toxicity of fluopicolide to earthworms. In this study, in order to evaluate the chronic toxicity of fluopicolide to earthworms, the levels of reactive oxygen species (ROS) and malondialdehyde (MDA), the activities of superoxide dismutase (SOD), catalase (CAT), and glutathione S-transferase (GST), and DNA oxidative damage (8-hyoxy-2-deoxyguanosine content) in earthworms were measured at 7, 14, 21, and 28 days after exposure to different concentrations (0, 0.1, 0.5, 1, 2.5, 5, and 10 mg/kg) of fluopicolide. In most treatment groups, the ROS levels increased significantly 7 days after exposure and then decreased gradually with an increase in exposure time, a certain dose–effect relationship. The antioxidant enzymes’ activities (SOD and CAT) in most treatment groups were activated, showing an increasing trend at first and then a decreasing trend; however, the CAT activity in the high-concentration treatment group was inhibited 21 days after exposure. The GST activity and MDA content showed an increasing trend at first and then a decreasing trend, which was dependent on the dose. As a biomarker of DNA damage, the 8-OHdG content was positively correlated with the concentration of fluopicolide. The results showed that a low dose of fluopicolide could cause oxidative stress and DNA damage in earthworms.

## 1. Introduction

Fluopicolide (2,6-dichloro-N-[(3-chloro-5-trifluoromethyl-2-pyridyl) toluene] benzamide) is a new benzamide fungicide developed by Bayer Crop Science Company with a unique action mechanism [1,2]. It is mainly used to prevent and control oomycete diseases such as downy mildew, late blight, epidemic disease, damping-off disease, etc. [3,4,5]. It has protective and broad-spectrum therapeutic effects [6]. Fluopicolide is widely used because of its unique mode of action, mainly acting on the cell membrane and specific proteins between cells to induce bactericidal activity and has no cross-resistance with other fungicides [7,8]. However, with the global widespread use of fluopicolide, people are gradually paying attention to its polluting potential after it has entered the environment [9]. 

The acute toxicity data of fluopicolide to some environmental organisms from the National Environmental Protection Agency of the United States show that fluopicolide is moderately toxic to *Brachydanio rerio*, *Scenedesmus obliquus*, and *Daphnia magna*; its acute toxicity to Lepomis macrochirus and Oncorhynchus mykiss is high [10]. It has been found that fluopicolide concentrations can also be enriched in B. rerio, which is a moderately enriched pesticide and has chronic toxicity to zebrafish juveniles, adults, and embryos [11,12]. However, there are few studies on the chronic toxicity of fluopicolide to earthworms.

Earthworms play an important role in the soil ecosystem via decomposition, nutrient mineralization, and soil structure improvement through burrowing, feeding, and other activities in the soil ecosystem [13,14,15]. Earthworms have no cuticle, so they are sensitive to pollutants. Exogenous pollutants remaining in the soil such as insecticides, fungicides, and heavy metals can easily enter the earthworm’s body through feeding or epidermal contact [16,17]. This fact can be used to detect the impact of low-dose pollutants on environmental safety [18]. Earthworms are regarded by the Organization for Economic Cooperation and Development (OECD) as a model organism for toxicological experiments [19,20] and as a biological indicator of chemical toxicity [21]. Therefore, earthworms are widely used to evaluate potential ecological risks caused by the ecotoxicological prediction of pesticides [22,23].

Biochemical reactions to environmental stress in organisms are sensitive and reproducible, and biotoxicity monitoring can be used for early environmental pollution warnings. Many enzyme activities are considered biomarkers of environmental pollution [24]. Reactive oxygen species (ROS) can be produced in organisms in polluted environments. Under normal circumstances, the production and removal of ROS are in dynamic equilibrium, but when organisms are stimulated by pollutants, ROS will accumulate in large quantities in vivo and react with lipid molecules to cause oxidative damage [25,26]. Malondialdehyde (MDA), as the final product of the oxidation reaction between reactive ROS and lipids, can reflect the degree of oxidative damage to cells from the side [17]. Reactive ROS scavenging in organisms mainly depends on antioxidant enzymes such as superoxide dismutase (SOD), catalase (CAT), and glutathione s-transferase (GST). Therefore, some oxidative damage is also attributed to a change in antioxidant enzyme activity [27,28].

In this experiment, we explored the effects of oxidative stress and DNA damage caused by a low dose of fluopicolide to earthworms by measuring ROS, SOD, CAT, GST, MDA, and 8-OHdG, and evaluated the potential ecological risk of fluopicolide to soil organisms [29,30,31].

## 2. Materials and Methods

### 2.1. Chemicals, Earthworms, and Soil

Fluopicolide (97% purity) was purchased from Bayer Aktiengesellschaft. All other chemicals and solvents were of analytical purity and were purchased from Shanghai Biotech Co., Ltd. (Shanghai, China), Beijing Solabo Technology Co., Ltd. (Beijing, China), and Tianjin Kaitong Chemical Reagent Co., Ltd. (Tianjin, China).

*E. foetida* was purchased from an earthworm farm in Jurong, Jiangsu, China. Under laboratory conditions, the earthworms were acclimatized for 2 weeks at 20 ± 1 °C. Healthy individuals (weights between 400 and 500) with fully developed clitella were selected for the formal experiment. The artificial soil used in the experiment was prepared with 10% sphagnum peat moss, 20% kaolin clay, and 70% quartz sand, which was mixed evenly, and each sample weighed 500 g. The pH of the soil was adjusted to 6 ± 0.5 by adding CaCO_3_ [20].

### 2.2. Experimental Design

The concentrations used in this study were determined according to the reported residual amount of fluopicolide in the soil. When the dosage is twice the recommended field dosage, the initial concentration of fluopicolide in the soil is approximately 2.5 mg/kg [32]. Therefore, in this experiment, the reagent concentrations were 0, 0.1, 0.5, 1, 2.5, 5, and 10 mg/kg. A standard working solution of fluopicolide was prepared with acetone as a solvent, and 5 mL of fluopicolide solution was added to 50 g of artificial soil and then fully mixed. After the acetone evaporated, this soil was mixed evenly with the remaining artificial soil. The mixed soil was transferred to 1 L beakers and the water content was adjusted to 35% of the total weight [33]. Ten pre-cultured earthworms were placed in each beaker. After the earthworms had buried into the soil, 5 g of cow dung (5 g per week) was placed on the soil surface and the beakers were covered with a plastic film with holes. Each treatment was repeated 3 times. These beakers were stored at 20 ± 1 °C under a 12 h light and 12 h dark cycle. On the 7th, 14th, 21st, and 28th days, two earthworms were taken out from each treatment and placed in a Petri dish with wet filter paper and depurated overnight to excrete intestinal contents.

### 2.3. Preparation of Earthworms’ Homogenates and Mitochondrial Suspension

In the preparation of earthworms’ homogenates, after washing and drying earthworms that spit mud overnight, earthworms were weighed on the balance and then placed into a homogenizer. Precooled 0.05 mol·L^−1^ potassium phosphate buffer (1:9, *w*/*v*, pH 7.8) was added to the 50 mL glass homogenizer and homogenized thoroughly on ice until the liquid in the homogenizer was uniform. The homogenates were centrifuged at 9000× *g* for 10 min, and the supernatant was centrifuged at 9000× *g* for 5 min. The supernatant was stored at 4 °C for later use.

In the preparation of earthworm mitochondrial suspension, after washing and drying earthworms that spit mud overnight, earthworms were placed into a homogenizer and 0.1 mol·L^−1^ of phosphate-buffered solution (PBS pH 7.4) was added, and homogenized at 4 °C. The homogenates were centrifuged at 1000× *g* at 4 °C for 10 min, and the supernatant was centrifuged at 2000× *g* at 4 °C for 20 min. The precipitate was resuspended with PBS and stored at 4 °C for later use.

### 2.4. Oxidative Stress

#### 2.4.1. Determination of ROS Levels

The level of ROS was determined by the dichloride fluorescein DCHF-DA method [34]. A total of 190 μL of the mitochondrial suspension was mixed with 10 μL of the DCHF-DA solution, and the mixture was incubated for 30 min at 37 °C. The reaction was terminated by adding 200 μL of a hydrochloric acid solution (1 mol·L^−1^), and the OD value was determined by fluorescence spectrophotometry at a 485 nm excitation wavelength and 538 nm emission wavelength. The results were expressed as fluorescence intensity per milligram of protein.

#### 2.4.2. Determination of SOD, CAT, and GST Activity

According to the WST-1 method, SOD activity was determined by using a SOD determination kit. The experiment consisted of a control hole, a control blank hole, a determination hole, and a determination blank hole. According to the operation table, earthworm homogenates, distilled water, the enzyme working solution, enzyme diluent, and the substrate application solution were added to the corresponding holes and incubated at 37 °C for 20 min, and the absorbance at 450 nm was recorded using a microplate reader. One unit (U) of SOD activity was defined as the corresponding enzyme amount when the SOD inhibition rate reached 50% in this reaction system. The result was expressed as U/mg of total protein.

According to the visible light method, CAT activity was determined by using a CAT test box. There was a control tube and a measuring tube in the experiment. According to the operation table, earthworm homogenates and the reagent were added to the corresponding tubes, and the absorbance at 405 nm was recorded using a microplate reader. One unit (U) of CAT activity was defined as the amount of H_2_O_2_ decomposed by 1 μmol per second per milligram of tissue protein. The result was expressed as U/mg of total protein.

GST activity was determined by using a glutathione-s transferase assay kit. The experiment was divided into two parts: An enzymatic reaction and a chromogenic reaction. After the chromogenic reaction, the mixture was placed at room temperature for 15 min and the absorbance at 412 was recorded using a microplate reader. The activity unit (u) of GST was described as every milligram of tissue protein that reacts at 37 °C for 1 min, and the concentration of GSH in the reaction system was reduced by 1μmol·L^−1^ as one enzyme activity unit after deducting the non-enzymatic reaction. The result was expressed as U/mg of total protein.

#### 2.4.3. Determination of MDA Activity

According to the TBA method, MDA activity was determined by using an MDA test box. The mixed solution (reaction reagent and earthworm homogenates) was placed in a water bath at 95 °C for 40 min, then cooled by running water and centrifuged at 4000 r·min^−1^ for 10 min and the supernatant was removed. The absorbance at 532 nm was recorded using a microplate reader.

### 2.5. DNA Damage

The content of 8-OHdG was determined by using an earthworm 8-hydroxydeoxyguanosine (8-OHdG) ELISA kit. Earthworm tissue homogenates and reagents were added to a 96-well plate. After the reaction, the absorbance at 450 nm was recorded using a microplate reader. Standards were used to draw standard curves. Then, the 8-OHdG content in the sample was calculated.

### 2.6. Determination of Protein Content

According to the BCA method, the protein content in earthworm tissue homogenates was determined by a BCA protein concentration determination kit. Earthworm tissue homogenates and reaction reagents were added to a 96-well plate. The 96-well plate was heated at 37 °C for 30 min, and the absorbance at 562 nm was recorded using a microplate reader. Protein standards were used to draw a standard curve. Then, the protein content in the sample was calculated.

### 2.7. Statistical Analysis

SPSS 20.0 was used to analyze one-way variance between the treatment group and the control group (*p* < 0.05). A two-factor analysis of variance (ANOVA) analyzed the concentration, time, and interaction of biochemical reactions. The results of the one-way ANOVA were statistically analyzed and plotted in Sigmaplot 14.0. LSD tests were used to examine the differences between treatment groups and control groups (the significance level was *p* < 0.05). All experimental results are expressed as means with standard deviation (SD).

## 3. Results

### 3.1. Oxidative Stress

#### 3.1.1. ROS Levels

**Figure 1:**
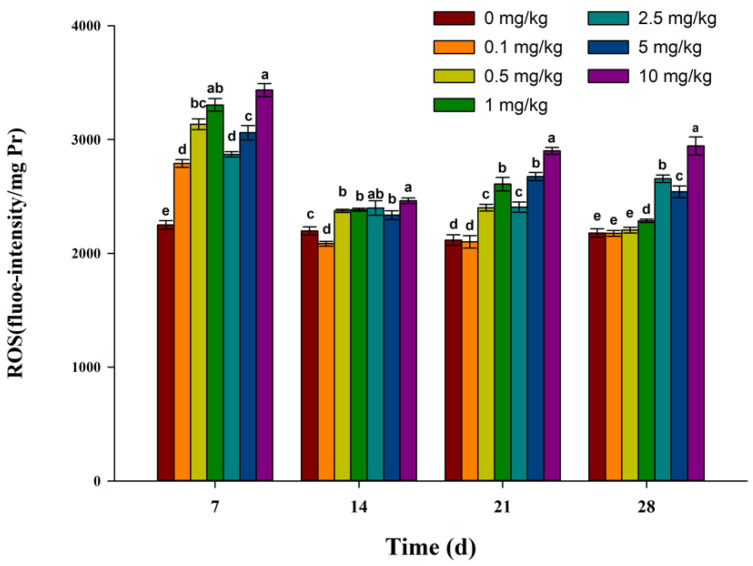
Effect of fluopicolide on the ROS levels in earthworms. The data are presented as means ± SD (*n* = 3). Different letters above the columns indicate significant differences between treatments at the *p* < 0.05 level.

The results of ROS levels are shown in Figure 1. The ROS levels of each treatment group were significantly higher than those of the control group after exposure for 7 days. Except for the 0.1 mg/kg treatment group, the ROS levels of other treatment groups were higher than that of the control group after exposure for 14 days. The ROS levels increased with an increase in the fluopicolide concentration, except for the 2.5 mg/kg treatment group after exposure for 21 days. Additionally, there was no difference between the 0.1 and 0.5 mg/kg treatment groups and the control group after exposure for 28 days. During the experimental period, the ROS level in earthworms exposed to the highest concentration (10 mg/kg) was significantly higher than in other treatment groups. In addition, comparing the exposure time, on the 7th day, the ROS level of each treatment group was higher than other exposure times.

#### 3.1.2. SOD Activity

**Figure 2:**
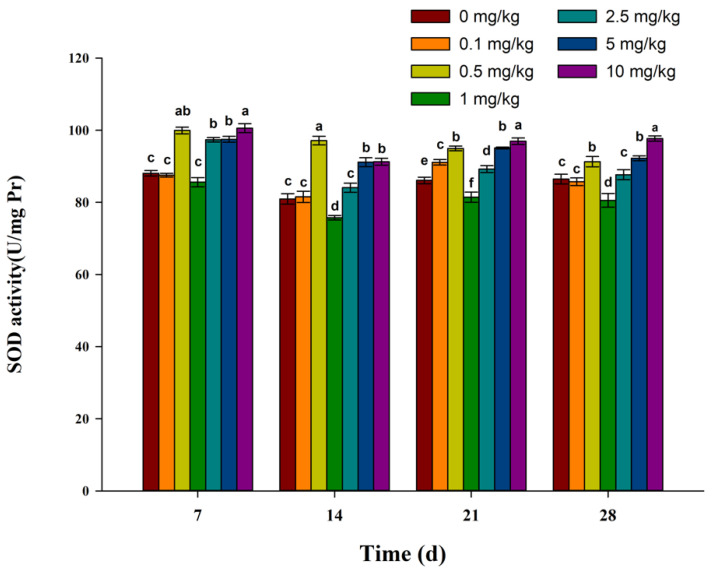
Effect of fluopicolide on the SOD activity of earthworms. The data are presented as means ± SD (*n* = 3). Different letters above the columns indicate significant differences between treatments at the *p* < 0.05 level.

The SOD activities in earthworms after exposure to fluopicolide are shown in Figure 2. The SOD activity in the 1 mg/kg treatment group was significantly lower than that in the control group on days 14, 21, and 28. The SOD activities in the 0.5, 2.5, 5, and 10 mg/kg treatment groups were significantly higher than those in the control group for all exposure times.

#### 3.1.3. CAT Activity

**Figure 3:**
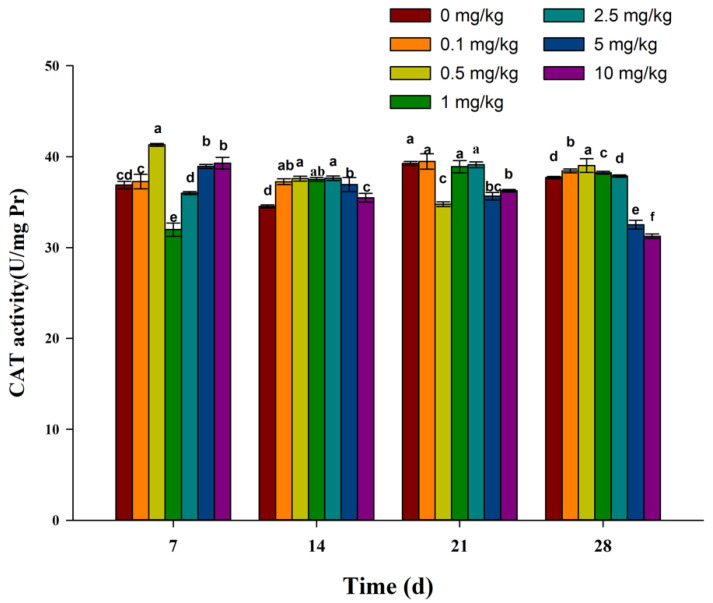
Effect of fluopicolide on the CAT activity of earthworms. The data are presented as mean ± SD (*n* = 3). Different letters above the columns indicate significant differences between treatments at the *p* < 0.05 level.

With the increase in exposure time, the CAT activity in 0.1, 1, and 2.5 mg/kg treatment groups increased at first and then decreased. The CAT activity in the 0.5 mg/kg treatment group decreased at first and then increased. The CAT activity in the 5 mg/kg and 10 mg/kg treatment groups decreased with the increase in exposure time. Compared with the control group, the CAT activity in the 1 mg/kg and 2.5 mg/kg treatment groups decreased 7 days after exposure, while the CAT activity in other concentration treatment groups increased. Fourteen days after exposure, the CAT activity increased significantly in all treatment groups. Twenty-one days after exposure, the CAT activity in 0.5 mg/kg, 5 mg/kg, and 10 mg/kg treatment groups decreased significantly, and there was no difference between the other treatment groups and the control group. Twenty-eight days after exposure, the CAT activity in 5 mg/kg and 10 mg/kg treatment groups decreased significantly compared to the control group, while the CAT activity in other treatment groups increased compared to the control group (Figure 3).

#### 3.1.4. GST Activity

**Figure 4:**
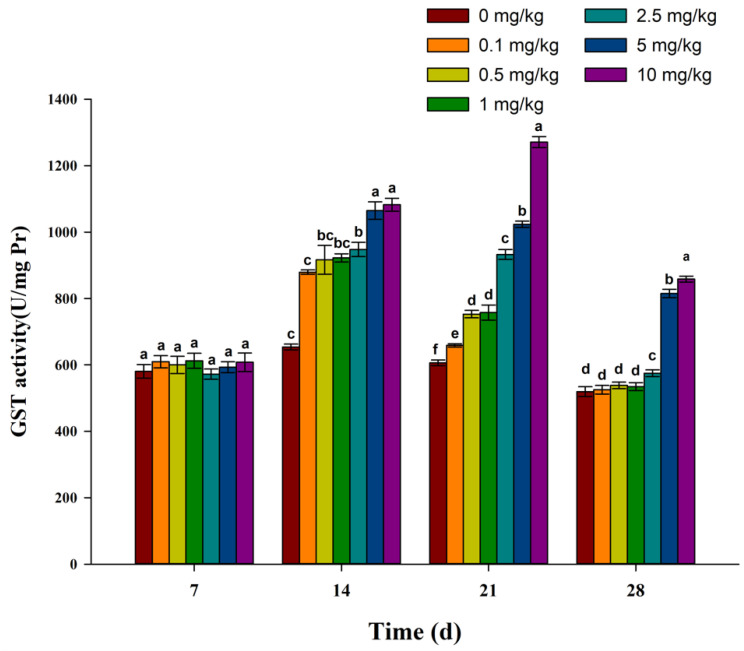
Effect of fluopicolide on the GST activity of earthworms. The data are presented as means ± SD (*n* = 3). Different letters above the columns indicate significant differences between treatments at the *p* < 0.05 level.

As shown in Figure 4, with the increase in exposure time, each treatment group showed a trend of increasing at first and then decreasing. Seven days after exposure, there was no difference in GST activity between each treatment group and the control group. Compared with the control group, the GST activity in each treatment group increased significantly on the 14th, 21st, and 28th day of exposure, and the GST activity was positively correlated with the treatment concentration.

#### 3.1.5. MDA Concentration

**Figure 5:**
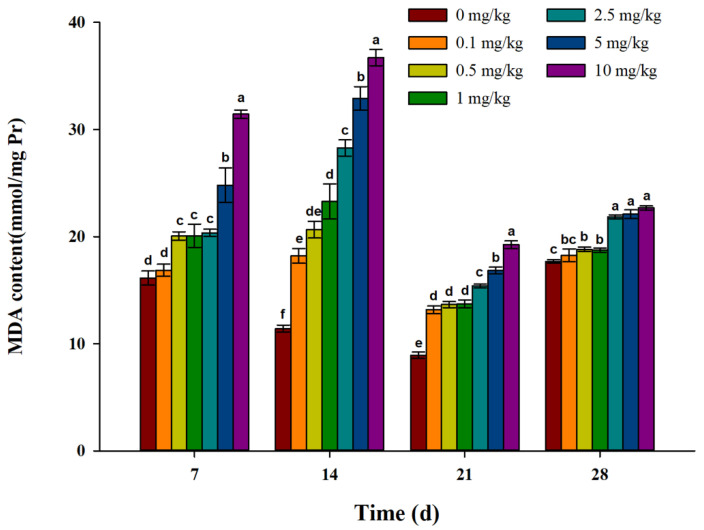
Effect of fluopicolide on the MDA content in earthworms. The data are presented as the means ± SD (*n* = 3). Different letters above the columns indicate significant differences between treatments at the *p* < 0.05 level.

The results of the MDA concentration are shown in Figure 5. The MDA concentration in each treatment group was significantly higher than that in the control group 7 days after exposure, and the levels were positively correlated with the treatment concentrations. On the 14th and 21st days after exposure, the MDA concentration in each treatment group was significantly higher than that in the control group, and the MDA concentrations were positively correlated with the treatment concentrations, with the highest MDA concentration in the 10 mg/kg treatment group. Twenty-eight days after exposure, the MDA concentration in the 0.1 mg/kg treatment group was not significantly different from that in the control group, while the MDA concentrations in other treatment groups were significantly higher than that in the control group, showing a certain concentration dose relationship.

### 3.2. DNA Damage

**Figure 6:**
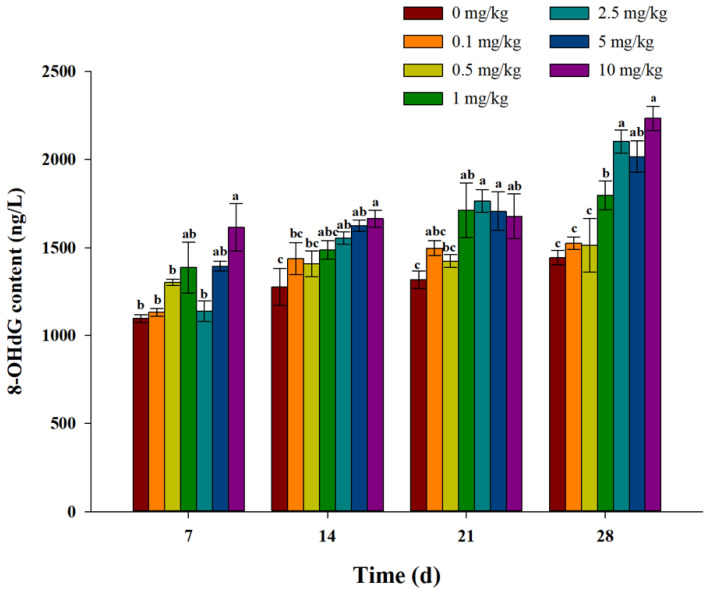
Effect of fluopicolide on the 8-OHdG content of earthworm. The data are presented as means ± SD (*n* = 3). Different letters above the columns indicate significant differences between treatments at the *p* < 0.05 level.

As shown in Figure 6, after exposure to fluopicolide for 7 days, the content of 8-OHdG in each treatment group was higher than in the control group. With the increase in exposure time, the content of 8-OHdG in each treatment group gradually increased, and the content of 8-OHdG was positively correlated with the concentration of fluopicolide. After exposure to fluopicolide for 28 days, the content of 8-OHdG in each treatment group was significantly higher than that in the control group, and the content of 8-OHdG in the 10 mg/kg treatment group was the highest.

### 3.3. Two-Way Crossed ANOVA of Biochemical Response

Among all the biomarkers evaluated, there was a significant correlation between fluopicolide concentration and exposure time. The analysis of concentration, exposure time, and concentration–time interaction showed significant differences in biomarkers of earthworms under concentration, exposure time, and concentration–time interaction (Table 1). Therefore, the interaction between concentration and exposure time significantly changed the biomarkers of oxidative stress and DNA damage in earthworms.

The effect of fluopicolide at different concentrations and with different exposure times on biomarkers of earthworms showed that there were significant differences in ROS level, SOD activity, CAT activity, GST activity, MDA content, and 8-OHdG content at different concentrations and exposure times (*p* < 0.05) (Table 2).

## 4. Discussion

In this study, we studied the oxidative stress and DNA damage of *E. foetida* for 28 days after exposure to fluopicolide. Through a literature review, it was found that there were no studies on the potential ecological risk of fluopicolide to earthworms in soil up to now. Therefore, it is necessary to comprehensively analyze the sub-chronic toxicity (oxidative stress and DNA damage) of fluopicolide to earthworms to provide a reference for the future application of fluopicolide in agriculture.

### 4.1. Oxidative Stress

ROS constitute the intermediate product produced by normal oxidative metabolism in vivo and are mainly produced by mitochondria. ROS mainly include superoxide anions (O^2-^), hydrogen peroxide (H_2_O_2_), hydroxyl radicals (OH^-^), and nitric oxide [35]. Under normal circumstances, the production and elimination of reactive oxygen species in organisms are in dynamic equilibrium. Under the stress of the external environment, this balance in the body is destroyed, which leads to reactive oxygen species accumulation. Due to the active chemical properties of reactive oxygen species, extensive accumulation will cause damage to nucleic acids, lipids, and proteins [36].

In this study, the ROS level of the vast majority of treatment groups increased significantly compared to the control group, and the highest concentration of ROS was higher than that of other treatment groups. This indicated that the dynamic balance between the production and elimination of ROS in earthworms was broken by the introduction of fluopicolide, causing ROS accumulation in cells. On the 14th day, the level of ROS in earthworms decreased, and on the 21st and 28th days, the level of ROS in earthworms in the low-concentration treatment group recovered to the control level, while the level of ROS in earthworms in the high-concentration treatment group increased. This may be due to the combined action of the antioxidant enzyme and detoxification enzyme, leading to the elimination of oxygen accumulated in earthworms. In the high-concentration treatment group, the activities of antioxidant enzymes and detoxification enzymes decreased in the later stage, which possibly led to a gradual accumulation of ROS in cells. Ma et al. [28], in a toxicological study of antioxidant defense systems and DNA damage to earthworms caused by pyrrolidine, concluded that ROS levels in earthworms first increased and then decreased to the control level under low-concentration treatments, while ROS levels were higher than that of the control group under high-concentration treatments. These results are consistent with the current research.

Antioxidant enzymes in organisms mainly include SOD, CAT, peroxidase (POD), etc. SOD is a key enzyme in antioxidant systems and can convert superoxide radicals into oxygen and hydrogen peroxide. CAT can convert hydrogen peroxide into nontoxic water and oxygen to remove excessive hydrogen peroxide in earthworms in order to avoid oxidative damage to cells caused by excessive hydrogen peroxide [37,38]. In this study, when earthworms were exposed to fluopicolide for 7 days, the SOD activity of earthworms was higher than that at other time points. On the 14th day, SOD activities in earthworms decreased, which may be related to excessive ROS production caused by fluopicolide. On the 21st and 28th days, SOD activities in earthworms increased again, which may be related to earthworms adapting to the existing ROS level. Compared with the control group, the CAT activity in the 1 mg/kg treatment group decreased significantly at 7 days after exposure, while the CAT activity in other concentration treatment groups increased. Fourteen days after exposure, the CAT activity increased significantly in all treatment groups. After 21 and 28 days, the CAT activities in the 5 mg/kg and 10 mg/kg treatment groups were significantly lower than that in the control group, which may be related to excessive ROS, produced by fluopicolide exposure, inhibiting CAT activity [39]. Peroxidase (POD) is also an important antioxidant enzyme in the human body, as it can also remove excessive H_2_O_2_. Therefore, it is still worth further discussion as to whether the changes in SOD activity and CAT activity are affected by the changes in peroxidase activity.

### 4.2. Detoxification Effects

GST is an important detoxification enzyme in organisms and has dual functions of detoxification and in vivo peroxide scavenging [40,41]. GST can catalyze the combination of glutathione with various substrates, and it also plays a key role in the antioxidant system [42]. In this study, with an increase in exposure time, GST activities in each treatment group showed an increasing trend at first and then a decreasing trend. GST was activated from 7 to 28 days and had an obvious dose effect. These results indicate that fluopicolide may increase GST activity, thus detoxifying harmful compounds and inhibiting ROS oxidative damage [30]. From 21 days to 28 days, the decrease in GST activity may be due to the degradation of fluopicolide or the action of antioxidant enzymes in earthworms. The reasons for these changes need further study.

### 4.3. Lipid Peroxidation

MD is the final product of lipid peroxidation caused by free radicals in organisms, and its content indirectly reflects the severity of cell attack by free radicals [43]. At present, MDA has been used as a biomarker to evaluate the oxidative damage of earthworms in many studies [14,44]. In this study, the MDA content in earthworms in the treatment groups was significantly higher than that in the control group on the 7th to 21st day, indicating that lipid peroxidation occurred in earthworms under the action of fluopicolide. The MDA content reached its highest value at 14 days and then gradually decreased. On the 28th day, the MDA content in low-concentration treatment groups (0.1, 0.5, and 1 mg/kg) recovered to the level of the control group. This may be due to accumulated ROS being eliminated by antioxidant enzymes, which reduced the oxidative stress in earthworms, resulting in a decrease in MDA content. In addition, it may be that the degradation of fluopicolide and the repair function of its own cells led to a decrease in MDA content.

### 4.4. DNA Damage

When organisms are stressed and ROS accumulate in large quantities, the accumulated ROS will cause oxidative and DNA damage to organisms [45]. 8-OHd is an oxidative adduct produced by ROS attacking the eighth carbon atom of the guanine base in DNA molecules. The detection of 8-OHdG levels can reflect the degree of oxidative damage and repair in vivo and the correlation between oxidative stress and DNA damage. 8-OhdG has become the most commonly used biomarker in DNA oxidative damage detection [31,46,47,48]. In this study, the content of 8-OhdG in each treatment group was significantly higher than that in the control group throughout the experiment. In addition, the 8-OhdG content in each treatment group increased with the increase in exposure time, and the 8-OhdG content was essentially positively correlated with the concentration of fluopicolide. These results indicate that earthworm DNA was damaged by the action of fluopicolide to a certain extent, and the higher the concentration of fluopicolide, the more serious the oxidative damage was. Zhao et al. [31] found that N-ethyl perfluorooctane sulfonamidoethanol could enhance the DNA damage of earthworm coelomic cells via oxidative stress.

## 5. Conclusions

In this study, the effects of oxidative stress and DNA damage on earthworms caused by fluopicolide were studied by measuring enzyme activity, MDA, ROS, and 8-OHdG content. It was found that fluopicolide induced oxidative stress in earthworms, which led to the accumulation of ROS in earthworms. The accumulation of ROS led to changes in antioxidant enzyme activities (SOD and CAT) and MDA contents, showing a certain dose–effect relationship. In addition, the 8-OHdG content in earthworms increased with the increase in fluopicolide concentration, which indicates that fluopicolide caused damage to earthworm DNA. Generally speaking, under fluopicolide stress, earthworms underwent oxidative stress and DNA damage. This study provides new insights into the toxicological effects of fluopicolide on earthworms and the ecological risks for organisms in soil ecosystems.

## Figures and Tables

**Table 1:** Two-way ANOVA results for biomarkers in earthworms exposed to fluopicolide.

Biomarkers	Dose			Time			Dose × Time		
	df	F	*p*	df	F	*p*	df	F	*p*
ROS level	6	218.490	0.000 *	3	534.776	0	18	28.763	0.000 *
SOD activity	6	224.806	0.000 *	3	120.681	0	18	9.157	0.000 *
CAT activity	6	43.998	0.000 *	3	23.419	0	18	71.759	0.000 *
MDA content	6	199.306	0.000 *	3	278.893	0	18	22.857	0.000 *
GST activity	6	258.273	0.000 *	3	782.605	0	18	46.698	0.000 *
8-OHdG content	6	19.701	0.000 *	3	44.729	0	18	2.640	0.03 *

Note: F stands for significant test for all variables; df stands for degree of freedom; * stands for a significant effect of fluopicolide dose, time, and dose × time (*p* < 0.05).

**Table 2:** Results of post hoc testing by LSD after two-way ANOVA for biomarkers in earthworms exposed to fluopicolide.

Biomarkers		Dose (mg/kg)						Time (day)			
	0	0.1	0.5	1	2.5	5	10	7	14	21	28
ROS level	f	e	d	b	c	b	a	a	c	b	b
SOD activity	d	d	a	e	c	b	a	a	d	b	c
CAT activity	c	a	a	d	b	e	f	a	b	a	b
MDA content	f	e	d	d	c	b	a	b	a	d	c
GST activity	f	e	d	d	c	b	a	d	a	b	c
8-OHdG content	c	c	c	b	b	ab	a	d	c	b	a

Note: Different letters indicate significant differences between different doses or time at the *p* < 0.05 level.

## Data Availability

The data presented in this study are available on request from the corresponding author.
